# Effects of a Web-Based Intervention for Adults With Chronic Conditions on Patient Activation: Online Randomized Controlled Trial

**DOI:** 10.2196/jmir.1924

**Published:** 2012-02-21

**Authors:** Michael Solomon, Stephen L Wagner, James Goes

**Affiliations:** ^1^Point-of-Care PartnersCoral Springs, FLUnited States; ^2^Division of Medical Education and ResearchCarolinas Healthcare SystemCharlotte, NCUnited States; ^3^Department of Health SciencesWalden UniversityMinneapolis, MNUnited States

**Keywords:** Chronic care, health information technology, patient activation, randomized controlled experiment, self-management, Web-based intervention

## Abstract

**Background:**

With almost one-half of Americans projected to have at least one chronic condition before 2020, a vital role of the health care system is to develop informed, engaged individuals who are effective self-managers of their health. Self-management interventions (SMIs) delivered face-to-face or by telephone (traditional SMIs) are associated with improved self-management knowledge, skills, and self-efficacy, which are expressed by the composite construct of patient activation, a predictor of health outcomes. Web-based interventions to support self-management across the spectrum of chronic diseases have the potential to reach a broader population of patients for extended periods than do traditional SMIs. However, evidence of the effectiveness of Web-based interventions on patient activation is sparse. High-quality studies featuring controlled comparisons of patients with different chronic conditions are needed to explore the interaction of Web-based interventions and patient activation.

**Objective:**

To explore the effect of a Web-based intervention on the patient activation levels of patients with chronic health conditions, measured as attitudes toward knowledge, skills, and confidence in self-managing health.

**Methods:**

For this 12-week study, prospective participants were selected from the patient panel of a regional health care system in the United States. The 201 eligible participants were randomly assigned to two groups. Intervention group participants had access to MyHealth Online, a patient portal featuring interactive health applications accessible via the Internet. Control participants had access to a health education website featuring various topics. Patient activation was assessed pre- and posttest using the 13-item patient activation measure. Parametric statistical models (*t* test, analysis of variance, analysis of covariance) were applied to draw inferences.

**Results:**

The Web-based intervention demonstrated a positive and significant effect on the patient activation levels of participants in the intervention group. A significant difference in posttest patient activation scores was found between the two groups (*F*_1,123_ = 4.438, *P* = .04, *r* = .196). Patients starting at the most advanced development of patient activation (stage 4) in the intervention group did not demonstrate significant change compared with participants beginning at earlier stages.

**Conclusions:**

To our knowledge, this is the first study to measure change in patient activation when a Web-based intervention is used by patients living with different chronic conditions. Results suggest that Web-based interventions increase patient activation and have the potential to enhance the self-management capabilities of the growing population of chronically ill people. Activated patients are more likely to adhere to recommended health care practices, which in turn leads to improved health outcomes. Designing Web-based interventions to target a specific stage of patient activation may optimize their effectiveness. For Web-based interventions to reach their potential as a key component of chronic disease management, evidence is needed that this technology produces benefits for a sustained period among a diverse population.

## Introduction

Care for patients with chronic diseases consumes 78% of the total cost of a US health care system that has been slow to adapt to the complex needs of this growing population [1,2]. With almost one-half of Americans projected to have at least one chronic condition before the end of this decade [3,4], a vital role of the health care system is to provide the tools necessary for chronically ill patients to make informed decisions about their health care, and to solve the problems encountered daily from living with a chronic condition [5,6]. Self-management programs are designed to aid in this development by educating patients about their diseases, teaching skills to promote self-care behaviors, and fostering self-confidence in patients’ abilities to manage their disease [7-9]. As patients’ capabilities in these three areas improve, their level of patient activation, a measure of self-management capabilities, increases [10]. This paper describes the results of a study of change in patient activation when a self-management program delivered via the Internet is used. The findings inform innovators of chronic care programs on strategies for leveraging information technology to address the challenges of delivering self-management support services to a large and growing population of patients.

Patients who believe they have a responsibility to take an active role in making decisions about their health are central to effective chronic care management [11,12]. As the principal managers of their own care [5], activated patients are more likely to adhere to activities for controlling symptoms and the progression of their disease [13,14]. A person’s activation level indicates the extent of his or her self-management capabilities, encompassing knowledge, skills, and self-efficacy [10,15]. Development of these self-management capabilities precedes change in health behavior or status [16,17]. Thus, the construct of patient activation is a predictor of health process and outcomes measures [14,18] and is therefore a key indicator of the quality of chronic care management [10].

Activated patients strive to understand their conditions, viewing problems as challenges with the confidence that they can be solved [8,19]. Acquiring these attributes of self-management is a learning process, involving the acquisition of knowledge and problem-solving skills that enable an individual to confidently engage in decision making and actions to effectively manage their chronic health condition [13]. The developmental nature of patient activation is represented by four stages. At the earliest stage, individuals form beliefs that taking control of their health is important. As people progress through the second and third stages of patient activation, they develop the knowledge and skills to become increasingly active in self-managing their health. The most activated patients (stage 4) sustain self-management behaviors except when confronted with new or stressful situations [19]. Considering that patients are confronting different types of challenges at each stage of patient activation, researchers hypothesize that the most effective self-management interventions (SMIs) will feature designs targeting patients at a particular stage [13]. Despite the implications for designers of Web-based interventions for self-management, no research testing this theory appears in the literature.

Evidence suggests that SMIs delivered by face-to-face and telephonic modalities (ie, traditional SMIs) are associated with improvements in self-management knowledge and skills [17,20] and self-efficacy [21] among patients with various chronic conditions. Traditional SMI studies measuring change in the composite of these self-management capabilities—expressed as patient activation—show improvement in patient activation levels of chronically ill participants [13]. Thus, traditional SMIs demonstrate effectiveness in helping patients develop their self-management capabilities and therefore serve a vital role in the broader goal of improving health outcomes.

Use of information technology to deliver SMIs via the Internet has the potential to reach a broader population of chronically ill patients for extended periods of time when compared with traditional SMIs [9,22,23]. Web-based interventions (also referred to as Internet-based interventions) are applications accessed via a website by patients and are designed to improve understanding of a health condition, change health behavior, and enhance problem-solving skills [24,25]. Certain Web-based interventions are aimed at helping patients develop self-management capabilities and modify self-care behaviors to better deal with their chronic conditions [23,26,27]. The ensuing discussion of Web-based interventions refers to applications with self-management support as the central focus.

The health care community’s understanding of the value of Web-based interventions is inhibited by a dearth of high-quality studies [28], high variability in the effectiveness of different types of Web-based interventions [24], and mixed results of their effect on self-efficacy [28,29], a key component of patient activation. Few studies of Web-based interventions have explored the broader construct of patient activation (encompassing knowledge, skills, and self-efficacy), an area of inquiry that holds promise because of the symbiotic relationship between knowledge, skills, and self-efficacy in self-management performance [8,16]. Furthermore, the literature on Web-based intervention research is dominated by disease-specific interventions and measures, with scant evidence of the effectiveness of applications designed for use by populations with a broad spectrum of chronic diseases. More experimental studies featuring controlled comparisons of samples comprising patients with different chronic conditions are needed to better understand the interaction between Web-based interventions and the attributes of self-management performance constituting patient activation [30,31].

The few high-quality studies in the literature of Web-based interventions and their effectiveness in developing self-management capabilities suggest that these applications help patients to understand their role in managing health and the fundamental aspects of their chronic conditions. For example, Web-based interventions increase patients’ awareness of the need to be actively engaged in their health care [32]. A systematic review of randomized controlled trials revealed that Web-based interventions with self-management education modules had a significant and positive effect on patients’ knowledge of their chronic conditions [29], a component of patient activation.

Research on the effects of Web-based interventions on the self-efficacy of people with chronic conditions is limited and results are mixed. Although the effect of Web-based interventions on self-efficacy showed promise in a meta-analysis [29], an insufficient number of studies precluded any conclusions. Web-based interventions designed specifically for patients with chronic conditions such as diabetes or arthritis showed significant enhancement in self-efficacy levels [28,33,34] compared with a more generalized application targeting broader chronic disease populations, which did not demonstrate a significant effect [35]. Although more research is needed before conclusions should be drawn, these results suggest that Web-based interventions designed to target specific chronic diseases may increase self-efficacy; no evidence exists that applications targeting a diverse population of chronically ill patients influence this important element of patient activation.

Only one study of a Web-based intervention’s effect on the composite construct of patient activation of patients with a chronic condition appears in the literature. This randomized controlled trial reported a significant improvement in patient activation among patients with diabetes [33]. The study described in this paper builds on that research by evaluating the effect of a Web-based intervention on patient activation of patients living with different chronic conditions, including asthma, diabetes, and hypertension.

The aim of this randomized controlled trial was to evaluate change in self-management capabilities—expressed as patient activation—when a group of chronically ill patients were provided with online access to self-management materials. Specifically, we hypothesized that patients given access to a Web-based intervention designed to support self-management in the context of a person’s particular chronic disease would demonstrate positive change in patient activation levels compared with control group participants. A secondary hypothesis was tested within the intervention group. We hypothesized that patients beginning at a lower stage of patient activation development would demonstrate greater change in patient activation than would participants starting at higher stages.

## Methods

### Study Setting and Participants

Prospective participants were selected from the patient panel of Carolinas HealthCare System, a regional health care-delivery system in the southeastern United States. Adult patients who were seen by 300 physicians employed by Carolinas HealthCare System and with a diagnosis of asthma, hypertension, or diabetes constituted the sampling frame. We selected patients between ages 18 and 64 years, inclusive, with a diagnosis of one of the three conditions, and who had visited a participating physician in the past 2 years but not in at least 180 days. Patients with a chronic disease who have not visited their doctor in at least 6 months suggests nonadherence with recommended care guidelines and may indicate low patient activation. Employees of Carolinas HealthCare System were excluded. Selected patients were sent personalized invitations to participate. This study’s research protocol was approved by the Carolinas HealthCare System Research Review Committee, which did not require an external trial registration for this health behavior research. The institutional review boards of Walden University and Carolinas HealthCare System also approved the study.

**Figure 1:**
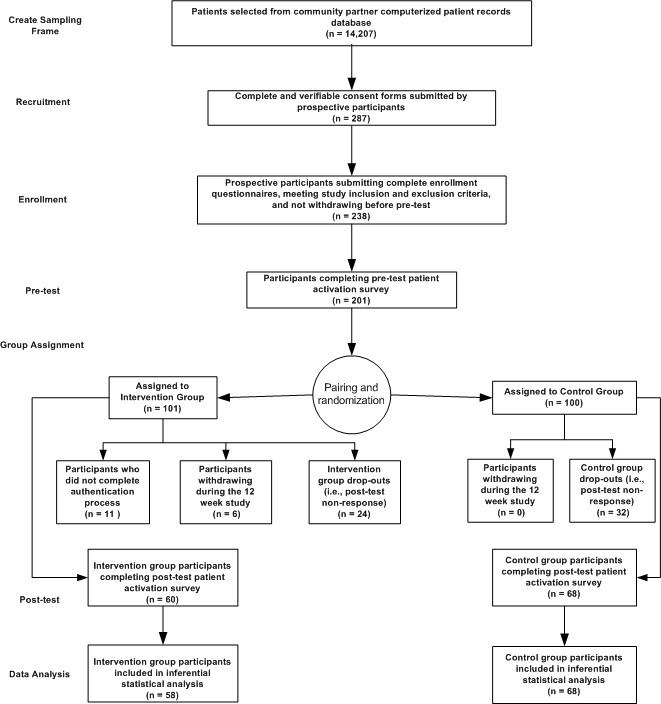
Flow of study participants.

All information used in this study for group assignments and data analysis was self-reported by participants and collected using a Web-based survey tool. Figure 1 shows the participants’ flow through the 12-week study.

Interested patients submitted online consents and enrollments. Patients meeting the eligibility criteria were placed in the pool of participants for the study. Participants were randomly assigned to the intervention or control group from pairs created by matching on adherence scores. We used a matching process because it helps to mitigate differences between the groups at baseline and strengthens the study’s statistical power [36]. The adherence scores were calculated based on participants’ responses to four items in the enrollment questionnaire related to a person’s adherence to self-management behavior. The two participants with the lowest adherence scores constituted the first pair and those with the highest scores, the last. Starting at an arbitrary point in the stack of pairs, we assigned one member of the pair to the intervention and the other to the control group.

On completion of the random assignments, participants were notified via express mail of their inclusion in the study, were presented with descriptive information regarding the self-management material that would be made available to them, and received instructions for accessing the pretest and study material on the Internet. Included in these directions was a unique and confidential login that controlled the specific participant’s access privileges to the appropriate intervention or control group material. Participants were not informed of their intervention or control status.

## Materials

We measured patient activation using the 13-item patient activation measure (PAM-13) [37]. The PAM-13 is designed to elicit responses from a person about his or her attitudes toward knowledge, skills, and confidence in self-managing health. The scale is based on the Guttman technique with items ordered according to level of difficulty. A 4-point Likert-type scale of response options ranging from strongly disagree to strongly agree is used to elicit endorsement of a particular statement. The PAM-13 statements are published by the developers elsewhere [37]. PAM-13 item responses result in total raw scores ranging from 13 to 52, which we converted to the linear interval scale of patient activation scores, ranging from 0 (lowest activation) to 100 (highest activation) [38]. Within this converted scale are cut-off minimum-point levels for each of the four stages of patient activation described earlier [13,19].

Psychometric analysis of the PAM-13 reported in the literature shows a measure with strong reliability and validity properties [37]. The unaltered PAM-13 was the pretest survey. The posttest survey included the PAM-13 plus an additional question about the participant’s visits to doctors during the study period.

The Web-based intervention used in this study was MyHealth Online, a personal health portal featuring a suite of interactive health applications. Effective Web-based interventions feature functions designed to change health behavior and improve patient–provider communications [24]. The MyHealth Online self-service and health education applications enable patients to manage their health care directly. MyHealth Online users can book doctors’ office appointments online, request prescription renewals, and view and pay their bills.

MyHealth Online’s interactive, multimedia health education modules are based on information therapy principles, with each online session designed to advance the user’s knowledge by providing evidence-based information on the patient’s specific condition, self-management guidelines, and options for problem solving and treatment [39]. Each week of the study, intervention participants received an email alerting them to the availability of the next in a progressive series of health education sessions specific to their chronic condition. Patients interact with the MyHealth Online health education modules at their own pace and decide the level of complexity of the material for a particular session. The self-directed design and 24-hour per day availability of MyHealth Online ensures that the information a patient needs to manage a particular problem is available when it is needed, facilitating decision making and behavior change [39].

If patients need more information, they can communicate online with their providers using the secure message function of MyHealth Online. Secure provider–patient email communication helps patients engage in their care and improves their access to information [40]. The software supporting MyHealth Online is supplied by GE Healthcare (Waukesha, WI, USA) and the health education applications are provided by Healthwise (Boise, ID, USA).

Control group participants were provided with access to a website hosting health education material on a variety of topics. In contrast to MyHealth Online, the materials available to control group participants were noninteractive and not prescriptive like the health education material provided to the intervention group. Participants were required to search topics to locate content of interest. Access to MyHealth Online or the control group’s website was granted to participants in the respective groups after they completed the pretest survey. All participants in the control and intervention groups were encouraged to continue their usual care during the study.

All participants in the study had access to the Carolinas HealthCare System website help desk for guidance on accessing and using the applications and to resolve technical problems. The intervention group participants were registered as end users of MyHealth Online and received no special services from the help desk. Support was limited to questions regarding the use and operation of the program. No self-management coaching was provided by any program resources. Intervention group participants received messages weekly via email reminding them to log in to MyHealth Online. Participants who fell below the desired threshold of participation (set at one log-in per week) received a message tailored to this condition, encouraging them to increase their participation and to contact the help desk if they required assistance to use the application. All control group participants received a message midway through the study reminding them to review the health education material and to contact the Carolinas HealthCare System help desk with any questions.

### Statistical Analysis

Except for the Rasch psychometric statistics [41], all statistical analysis was performed using PASW statistics release 18 programs (SPSS Statistics GradPack; IBM Corporation, Somers, NY, USA). We used Winsteps version 3.6 (Rasch Measurement Software, Chicago, IL, USA) to calculate the Rasch person reliability and infit statistics for assessing the PAM-13’s reliability and validity. Differences between the two groups were tested using the chi-square test of independence or Fisher exact test for categorical variables, and the *t* test for independent groups for pretest patient activation scores. These tests were also applied to assess between-group differences in the characteristics of patients who withdrew or dropped out of the study.

Interval-level data that were normally distributed resulted from this study, supporting the application of parametric models for the inferential tests. For testing the primary hypothesis, we evaluated the difference between groups in mean patient activation scores by applying analysis of covariance using the mean patient activation score at pretest as the covariate to reduce error variance. To test the second hypothesis and using a 2-step method, we analyzed change in patient activation scores between participants in the intervention group starting at different stages of patient activation. First, we divided intervention participants into three groups based on their stage of patient activation at the beginning of the study. We treated these groups as independent groups and applied 1-way analysis of variance to test for significance between groups. The 1-way analysis of variance is appropriate for comparing means between three groups but does not reveal the specific group differences underlying a significance difference [42]. To determine the specific groups (ie, baseline stage) demonstrating significant change, we conducted a post hoc test, using the Tukey honestly significant difference (HSD) method.

## Results

### Descriptive Characteristics

Table 1 presents demographic statistics for the control and intervention groups at the start of the study. The sample at baseline consisted of predominately non-Hispanic white persons between 45 and 64 years of age with a college degree. The sample consisted of slightly more women than men.

Table 2 shows health characteristics of the control and intervention groups at baseline. Most participants self-reported hypertension or diabetes as their main chronic condition.

**Table 1:** Demographic characteristics and between-group statistics at baseline.

Variable			Total sample (N = 201)		Control group (n = 100)		Intervention group (n = 101)		Test for difference	
			n	%	n	%	n	%	χ^2^	*P* value
**Age group (years)**									χ^2^_2_ = 0.5	.77
		25–44	36	18	16	16%	20	20%		
		45–54	66	33%	33	33	33	33%		
		55–64	99	49%	51	51%	48	47%		
**Gender**									χ^2^_1_ = 0.3	.62
		Male	96	48%	46	46%	50	50%		
		Female	105	52%	54	54	51	50%		
**Race**									χ^2^_1_ = 0.6	.43
		White	173	86%	88	88%	85	84%		
		Other	28	14%	12	12%	16	16%		
**Ethnicity**									χ^2^_1_ = 0.3^a^	.58
		Hispanic, Latino, or Spanish origin	12	6%	5	5%	7	7%		
		Other	188	94%	94	95%	94	93%		
**Education**									χ^2^_2_ = 0.2^a^	.90
		High school graduate or less	11	5%	5	5%	6	6%		
		Some college or trade school	66	33%	34	34%	32	32%		
		College graduate or more	123	62%	60	61%	63	62%		

^a^ One participant assigned to the control group did not respond to this item.

**Table 2:** Health characteristics and between-group statistics at baseline.

Variable			Total sample (N = 201)		Control group (n = 100)		Intervention (n = 101)		Test for difference	
			n	%	n	%	n	%	χ^2^	*P* value
**Main chronic condition**									χ^2^_3_ = 4.0^a^	.27
		Asthma	13	7%	7	7%	6	6%		
		Diabetes	43	22%	16	16%	27	27%		
		Hypertension	115	57%	60	60%	55	55%		
		Other	29	14%	17	17%	12	12%		
**Doctor visit in the past 6 months**									χ^2^_1_ = 2.2^b^	.13
		Yes	139	70%	65	65%	74	75%		
		No	60	30%	35	35%	25	25%		
**Have used health education classes, support groups, or materials from doctor**									χ^2^_1_ = 0.2	.68
		Yes	34	17%	18	18%	16	16%		
		No	167	83%	82	82%	85	84%		
**Reflecting on the past 6 months...I did different tasks and activities needed to manage my health condition so as to reduce my need to see a doctor**									χ^2^_1_ = 0.0	.94
		Disagree	53	27%	26	26%	27	27%		
		Agree	147	73%	73	74%	74	73%		

^a^ One participant assigned to the intervention group did not respond to this item.

^b^ Two participants assigned to the intervention group did not respond to this item.

At baseline no significant differences were found between the groups on any background variables, and the groups were not statistically different at the end of the study. Based on participants completing the study, there was no significant difference between the groups’ mean pretest patient activation scores. Thus, the two groups at posttest were not significantly different from when the trial started. Furthermore, we found no significant difference between the groups in the average frequency of office visits during the study. This result suggests that any difference in patient activation scores between the groups at posttest was not influenced by patients seeing their doctors.

The control and intervention groups experienced attrition rates of 32% (32/100) and 41% (41/101) respectively, by the end of the study (Figure 1). We found no significance difference between the dropouts of the two groups in mean pretest patient activation scores (*t*_71_ = .829, *P* = .41). Furthermore, the average pretest score of dropouts was not significantly different from that of participants who completed the study (*t*_197_ = –.951, *P* = .34). These results indicate that participants who completed the study did not differ from those who did not in terms of the dependent variable at pretest.

### Psychometric Properties of the PAM-13

The PAM-13 is based on the Rasch item response model [41]. Reliability of the PAM-13 is evaluated using the person reliability index; fit statistics are calculated to test construct validity of the measure [37]. The person reliability coefficient was a relatively high .83, showing a good spread of responses across the ordered items and expected endorsement patterns. Item and person infit statistics of .99 and 1.01, respectively, were well within the acceptable range [37]. In sum, the PAM-13 demonstrated strong psychometric properties in this study.

### Effect of the Web-Based Intervention on Patient Activation

The Web-based intervention demonstrated a positive and significant effect on the patient activation levels, on average, of the participants in the intervention group. Controlling for the pretest patient activation scores, we found a significant difference in posttest patient activation scores between the two groups (*F*_1,123_ = 4.438, *P* = .04, effect size *r* = .196). Both groups experienced an average increase in patient activation scores during the study (Table 3), prompting an examination of change scores to determine whether the improvement in the control group may have attenuated the between-group difference at posttest. The difference in the mean patient activation score from pretest to posttest of the control group (mean 2.04, SD 10.01) was not significant (*t*_67_ = 1.677, *P* = .10), whereas the intervention group showed a highly significant change (mean 5.967, SD 9.70, *t*_57_ = 4.683, *P* < .001). The positive and significant change in the intervention group’s patient activation reinforces the analysis of covariance results.

**Table 3:** Patient activation measure from pretest to posttest by group.

Group	n^a^	Patient activation score (pretest)		Patient activation score (posttest)		Analysis of covariance^b^	
		Mean	SD	Mean	SD	*F*_1,123_	*P* value
Control	68	66.89	10.94	68.93	12.28		
						4.438	.04
Intervention	58	65.33	14.17	71.30	13.74		

^a^ Group size (n) at posttest.

^b^ Analysis of covariance on 12-week posttest scores controlling for patient activation score at pretest.

### Baseline Patient Activation Stage and Change in Activation Scores

Prior to testing for differences in patient activation score changes between participants starting at each stage of patient activation, we combined patients at the first two stages into a single group to make this group’s size comparable with the others (Table 4). The 1-way analysis of variance revealed that the mean change in patient activation scores across the three groups was significantly different (*F*_2,55_ = 6.472, *P* = .003, effect size *r* = .436. Post hoc comparisons using the Tukey HSD test showed a significantly lower mean change score in the stage 4 group than in the combined stage 1–2 group (mean difference –8.457, 95% confidence interval –15.47 to –1.45; *P* = .01, effect size *d* = 0.45) and than among participants starting at stage 3 (mean difference –8.354, 95% confidence interval –15.06 to –1.64; *P* = .01, effect size *d* = 0.45). The difference in change scores between the stage 1–2 and stage 3 groups was nonsignificant (*P* = .999).

Despite beginning the study at the most advanced stage of patient activation, stage 4 group participants had the potential to substantially improve their patient activation scores. A ceiling effect was not evident in the stage 4 group, as 79% (22/28) of the participants began with activation scores between 68 and 80, sufficiently below the maximum score of 100, to experience a mean change in patient activation comparable with groups beginning at the earlier stages.

**Table 4:** Descriptive statistics for intervention group by patient activation stage at pretest.

Patient activation stage	n	Patient activation score (pretest)		Patient activation score (posttest)		Change in patient activation score	
		Mean	SD	Mean	SD	Mean	SD
1–2	14	46.11	4.42	56.19	9.50	10.08	7.87
3	16	61.38	3.85	71.35	11.35	9.98	10.64
4	28	77.2	7.72	78.82	10.32	1.62	8.26

## Discussion

To our knowledge, this is the first study to examine the effect of a Web-based intervention designed to target various chronic conditions on patient activation, a construct encompassing a person’s knowledge, skills, and self-efficacy to self-manage his or her health. It is also the first study to explore change in patient activation development stages among patients using a Web-based intervention. Results from this controlled trial suggest that a Web-based intervention targeting various chronic diseases and featuring the versatility to support self-management in the context of a person’s particular chronic condition may improve the level of patient activation, a measure of self-management capabilities.

We found a small but significant difference in posttest patient activation scores between the control and intervention groups. The small effect size found between the groups’ patient activation scores is consistent with other Web-based interventions demonstrating a significant effect on health behavior outcomes [24]. Within the intervention group, participants starting this 12-week study at the first three stages of patient activation improved their patient activation scores significantly compared with patients who started at the most advanced stage (4) of activation.

MyHealth Online is designed for use by patients with various health interests. Within this general-purpose architecture, applications are configurable to present content that targets a person’s specific chronic condition. For this study, parameters were set to provide messages and health education modules to an intervention group participant based on the main chronic disease self-reported during the enrollment process. SMIs that embed general self-management skills training within interactive disease-specific modules are more effective than didactic disease education alone in enhancing the self-efficacy component of the patient activation construct [43]. Building on this evidence, health education modules in MyHealth Online contain interactive exercises that present a self-management problem in the context of a specific disease (eg, adhering to a hypertension medication regimen). This approach resembles the disease-specific education of Web-based interventions shown to enhance the self-efficacy of patients with arthritis [44] and diabetes [33], and is in contrast to a general-purpose Web-based intervention [35], which did not show significant improvement in the self-efficacy component of patient activation. Thus, the present study suggests that, to improve patient activation, Web-based interventions designed for use by populations consisting of people with different chronic diseases should be designed to provide information therapy that is specific to a patient’s medical condition.

People in the first three stages of patient activation are forming beliefs about their role in personal health and starting to build confidence in their abilities to self-manage [13]. From a chronic care management perspective, a desirable goal is to develop patients’ self-management capabilities to the level reflected by the most advanced stage of patient activation. Patients with chronic conditions who are the most activated (ie, stage 4) are more likely to engage in self-management behaviors that are associated with improved clinical outcomes, including adhering to prescribed medication regimens, regularly testing glucose levels, and monitoring blood pressure [15]. The significant improvement in patient activation shown by intervention participants starting at the first three stages of patient activation suggests that MyHealth Online may have helped these patients become more capable of self-managing their health. Web-based interventions that aid the advancement of patients on the developmental continuum of patient activation contribute to an expanded population of patients who are able to actively manage their diseases—adhering to desirable health behaviors and knowing what steps to take when confronted with a condition-related incident (eg, diabetic hypoglycemia).

My Health Online appears to have aided primarily participants at the early stages of patient activation in gaining an understanding of their chronic conditions and developing the skills and self-confidence needed to better manage them. This apparent area of effectiveness of MyHealth Online is similar to findings from previous studies of the effects of Web-based interventions on the self-efficacy component of patient activation. Wangberg found that diabetic patients with the lowest baseline self-efficacy benefited the most from a Web-based intervention [45]. The nonsignificant change in stage 4 patients’ activation scores in the current study is comparable with the observation by Berman et al that patients with high baseline levels of the more granular attribute of self-efficacy did not show significant change as a result of a Web-based intervention [46].

Highly activated (ie, stage 4) patients lack confidence in their abilities to solve problems they have not previously encountered [13]. Despite room for improvement among participants beginning at stage 4 of patient activation in this study, they did not appear to gain substantive benefit from the SMI that was used. This outcome suggests that the self-service and interactive health education modules of MyHealth Online did not improve the self-confidence of participants who possessed the knowledge and skills to self-manage their health in routine situations. They may require a more sophisticated SMI with scenarios for how to solve various complex problems not frequently encountered. More research is needed to understand the specific needs of patients at the most advanced stage of patient activation.

The contrast in change in patient activation of people at different starting points of self-management capabilities affirms the assertion by Hibbard and colleagues [13] that different SMIs are needed to help people on their journey along the continuum of patient activation. Although more research is needed, evidence suggests that a Web-based intervention should be designed to specifically target either people with low levels of patient activation or those in the advanced stage of patient activation. A Web-based SMI with features similar to those of MyHealth Online may yield the most optimal results when used by patients in the earlier stages of patient activation. By recognizing the different stages of patient activation and targeting Web-based interventions to patients in the earlier stages, chronic care program designers and practitioners may derive tangible value from this technology as measured by improvements in quality metrics for adherence and health status.

### Strengths and Limitations

This study has several strengths and limitations. Important strengths were the rigorous randomized experimental design and posttest sample size (n = 128). The groups were statistically not different in terms of demographic characteristics and the dependent variable at baseline. Data met the assumptions for appropriate use of parametric tests. The dependent variable (patient activation score) was transformed to an interval-level scale, distributions of patient activation scores pre- and posttest were close to normal, variance of these scores shown by the two groups was sufficiently homogeneous, and the combined sample size exceeded the minimum needed to produce accurate results [42,47]. In addition, the PAM-13 demonstrated good psychometric properties.

Four limitations should be considered when interpreting the results. First, results may not be applicable to other populations and settings. Compared with the US population [4], the sample underrepresented people belonging to minority groups. Furthermore, 95% (189/200) of the study participants had attended college. This study follows a persistent pattern of research involving Web-based SMIs, which attract mostly college-educated, non-Hispanic, white participants [31,33,35,45,48,49]. Thus, the applicability of results from most research on Web-based interventions is inherently limited to a population willing and able to access the Internet. Further study is warranted to validate the results from this study with larger samples that are more representative of the US chronic disease population.

The second limitation is the unexplained reasons for the 41% attrition rate in the intervention group, a level higher than the 12%–25% reported for controlled trials involving Web-based interventions [44,46,50]. The six withdrawals and 24 dropouts may have been participants who experienced difficulties using the applications or had expectations for the Web-based intervention that were not met. These are problems reported by users of Web-based interventions in prior research [27,51]. The significance of usability or user expectations to this study’s level of attrition is unknown, as measuring user satisfaction was not a study objective. Furthermore, although the two groups maintained statistical equivalency on all demographic and health descriptive variables at posttest despite the attrition, attributes of the dropouts not measured in this study may have influenced the study’s outcomes.

Third, the scope of this research was limited to measuring change in patient activation when a Web-based intervention was used. The relative importance of specific functions and participants’ perceived value of the Web-based intervention were not assessed. A fourth limitation is the possibility that participants were influenced by agents external to the study. Participants were encouraged to receive usual care; no restrictions were placed on use of other support resources.

### Conclusion

Results from this study suggest that Web-based interventions have the potential to serve a vital role in health care providers’ efforts to enhance the self-management capabilities of the growing population of chronically ill people. The study’s outcomes reveal that the use of a Web-based intervention may result in more activated patients who are more proficient and self-confident in self-managing their chronic conditions. As a measure of self-management capabilities and a predictor of health outcomes, patient activation and its underlying components are mediators [52] in a causal chain of chronic care beginning with an SMI and ending with improved health outcomes. Activated patients are more likely to adhere to recommended health care practices, which in turn leads to improved health outcomes [12,53]. Thus, Web-based interventions that influence patient activation are an essential element of chronic disease management programs where success is measured in quality metrics such as patient adherence, functional status, or clinical effectiveness. Evaluating the effect of a Web-based intervention on patient activation provides program designers and practitioners with a more complete picture of the factors contributing to change in the health status of targeted patients.

As innovators work to transform health care to support the new “...era of chronic disease predominance” [11], they are encouraged to build on the discoveries from this and previous research to advance the industry’s understanding of the value of Web-based SMIs in activating patients to take control of their health. By providing self-management support services on demand, anytime, and from anywhere, Web-based interventions have the potential to be a transformative technology in the management of chronic diseases, delivering self-management services to large numbers of people with fewer human resources than traditional SMIs have. To turn this potential into reality, evidence is needed that Web-based interventions can produce benefits for a sustained period among a diverse population. More clarity of the value of Web-based self-management tools as scalable interventions will stimulate the investments necessary to accelerate adoption throughout all segments of the large and growing chronic disease population.

## Abbreviations

HSD: honestly significant difference
PAM-13: 13-item patient activation measure
SMI: self-management intervention

## Multimedia Appendix 1

CONSORT-EHEALTH checklist V1.6 [54].
