# Supplementary material for: Effects of a Web-Based Intervention for Adults With Chronic Conditions on Patient Activation: Online Randomized Controlled Trial
Source: J Med Internet Res. 2012 Feb 21;14(1):e32. doi: 10.2196/jmir.1924 (PMC3374536; doi:10.2196/jmir.1924)
Supplement: Supplementary file 1 [file jmir_v14i1e32_app1.pdf]

CONSORT-EHEALTH Checklist V1.6 Report

**Manuscript Number**	1924
**Date completed**	8/25/2011 14:50:29
**by**	Michael R Solomon

Effects of a Web-based intervention for adults with chronic conditions on patient activation: An online randomized controlled trial

TITLE

1a-i) Identify the mode of delivery in the title
"Effects of a Web-based intervention...."

1a-ii) Non-web-based components or important co-interventions in title
No non-web-based components or co-interventions used in the study.

1a-iii) Primary condition or target group in the title
"Effects of a Web-based intervention for adults with chronic conditions...."

ABSTRACT

1b-i) Key features/functionalities/components of the intervention and comparator in the METHODS section of the ABSTRACT
"Intervention group participants had access to MyHealth Online (MHO), a patient portal featuring interactive health applications accessible via the Internet. Control participants had access to a health education website featuring various topics."

1b-ii) Level of human involvement in the METHODS section of the ABSTRACT
Throughout the manuscript, the exclusive use of Web-based online tools is described.

1b-iii) Open vs. closed, web-based (self-assessment) vs. face-to-face assessments in the METHODS section of the ABSTRACT
"...prospective participants were selected from the patient panel of a regional health care system located in the United States.'

1b-iv) RESULTS section in abstract must contain use data
"The web-based intervention demonstrated a positive and significant effect on the patient activation levels of participants in the intervention group. A significant difference in posttest patient activation scores was found between the two groups ($F_{1,123} = 4.438$, $P = .037$, $r = .196$). Patients starting at the most advanced development of patient activation (stage four) in the intervention group did not demonstrate significant change compared to participants beginning at earlier stages. A strong relationship between MHO use and change in patient activation was found. Intervention participants logging into the web-based intervention at least 12 times during this study experienced a significantly larger change in patient activation scores compared to patients with a lower frequency of logins ($t_{56} = -2.862$, $P = .006$, $d = .764$)."

Attrition is discussed in the manuscript.

1b-v) CONCLUSIONS/DISCUSSION in abstract for negative trials
"This is the first known study to measure change in patient activation when a web-based intervention is used by patients with various chronic conditions. Results suggest that web-based interventions increase patient activation and have the potential to enhance the self-management capabilities of the growing population of chronically ill. To turn this potential into reality, evidence is needed that web-based interventions product benefits for a sustained period among a diverse population. "

INTRODUCTION

2a-i) Problem and the type of system/solution
"With almost one-half of Americans projected to have at least one chronic condition before the end of this decade [3,4], a vital role of the health care system is to provide the tools necessary for chronically ill patients to make informed decisions about their health care, and to solve the problems encountered daily from living with a chronic condition...."

"Use of information technology to deliver self-management interventions via the Internet has the potential to reach a broader population of chronically-ill patients for extended periods of time when compared to traditional SMIs..."

2a-ii) Scientific background, rationale: What is known about the (type of) system
"The health care community's understanding of the value of web-based interventions is inhibited by the dearth of high-quality studies, high variability in the effectiveness of different types of web-based interventions [23] and mixed results of their effect on self-efficacy [22], a key component of patient activation. Few studies of web-based interventions have explored the broader construct of patient activation, an area of inquiry that holds promise because of the symbiotic relationship between knowledge, skills, and self-efficacy in self-management performance [8,32]. Furthermore, the literature on web-based intervention research is dominated by disease-specific interventions and measures, with scant evidence of the effectiveness of applications designed for use by patients with a variety of chronic diseases. More experimental studies featuring controlled comparisons of patients with various chronic conditions are needed to better understand the interaction between web-based interventions and attributes of self-management encapsulated by patient activation [24]."

METHODS

3a) CONSORT
"The aim of this randomized controlled trial was to evaluate change in self-management capabilities – expressed as patient activation – when patients with a variety of chronic medical conditions are provided online access to self-management materials. Specifically, we hypothesized that patients given access to a web-based intervention designed to develop the self-management capabilities of patients with a variety of chronic medical conditions would demonstrate positive change in patient activation levels compared to control group participants. Two secondary, related hypotheses were tested within the intervention group. We hypothesized that patients beginning at a lower stage of patient activation development would demonstrate greater change in patient activation compared to participants starting at higher stages. The last hypothesis postulated that a relationship would exist between the frequency of use of the web-based intervention by patients in the intervention group and change in patient activation."

3b-i) Bug fixes, Downtimes, Content Changes
No substantive technical issues arose with the intervention or control materials during the course of the study. No enhancements were made, and no significant downtime was experienced during the 12-week study.

4a-i) Computer / Internet literacy
Although the online procedures required to sign up for the study were straightforward, they were designed to eliminate that segment of the study population that did not have access to the Internet and a basic level of literacy for using web-based computer applications.

4a-ii) Open vs. closed, web-based vs. face-to-face assessments:
"Prospective participants were selected from the patient panel of Carolinas Healthcare System (CHS), a regional health care delivery system located in the southeastern United States."

"Interested patients submitted online consents and enrollments."

4a-iii) Information giving during recruitment
CHC Research Review Committee and IRB required consent forms that described the study and its voluntary nature. Invitation letters (also approved by IRB) originated from the patient's doctor who described his/her support for the study.

4b-i) Report if outcomes were (self-)assessed through online questionnaires
"All information used in this study for group assignments and data analysis was self-reported by participants and collected using a web-based survey tool. "

4b-ii) Report how institutional affiliations are displayed
Invitations, web-based consent forms, intervention and control group materials all displayed the CHS logo.

5-i) Mention names, credential, affiliations of the developers, sponsors, and owners
"The software supporting MHO is supplied by GE Healthcare and the health education applications are provided by Healthwise."

5-ii) Describe the history/development process
MyHealth Online and Healthwise products were in commercial use prior to this study. No description of the development history was deemed essential to the outcomes of the study.

5-iii) Revisions and updating
MyHealth Online and Healthwise products were in commercial use prior to this study. No description of the development history was deemed essential to the outcomes of the study.

5-iv) Quality assurance methods
A pilot study was conducted previous to the trial to test the instruments and materials.

5-v) Ensure replicability by publishing the source code, and/or providing screenshots/screen-capture video, and/or providing flowcharts of the algorithms used

The intervention and control materials contain proprietary intellectual property from commercial vendors. Publishing source code, screen shots, etc. is not feasible.

5-vi) Digital preservation

The intervention and control materials contain proprietary intellectual property from commercial vendors. Publishing URLs, source code, screen shots, etc. is not feasible.

5-vii) Access

"Upon completion of the random assignments, participants were notified via express mail of their inclusion in the study, presented with descriptive information regarding the self-management material that would be made available to them, and received instructions for Internet access to the pre-test and study material. Included in these directions was a unique and confidential login which controlled the specific participant's access privileges to the appropriate intervention or control group material."

5-viii) Mode of delivery, features/functionalities/components of the intervention and comparator, and the theoretical framework

"The web-based intervention used in this study was MyHealth Online (MHO), a personal health portal featuring a suite of interactive health applications. Effective web-based interventions feature functions designed to change health behavior and improve patient-provider communications [23]. The MHO self-service and health education applications enable patients to engage directly in managing their health care. MHO users can book doctors' office appointments online, request prescription renewals, and view and pay their bills.

The interactive, multimedia health education modules are based on information therapy principles, with each online session designed to advance the user's knowledge by providing evidence-based information on the patient's specific condition, self-management guidelines, and options for problem-solving and treatment."

"Control group participants were provided access to a website hosting health education material on a variety of topics. In contrast to MHO, the materials available to control group participants were non-interactive and not prescriptive like the health education material provided to the intervention group. Participants were required to search topics to locate content of interest."

"The software supporting MHO is supplied by GE Healthcare and the health education applications are provided by Healthwise. "

5-ix) Describe use parameters

"Intervention group participants received messages weekly via e-mail reminding them to log in to MyHealth Online. Participants who fell below the desired threshold of participation (set at one log in per week) received a message tailored to this condition, encouraging them to increase their participation and to contact the help desk if they required assistance to use the application. All control group participants received a message midway through the study reminding them to review the health education material and to contact the CHS help desk with any questions."

5-x) Clarify the level of human involvement

"All participants in the study had access to CHS' web site help desk for guidance on accessing and using the applications and to resolve technical problems. The intervention group participants were registered as end-users of MyHealth Online and receive no special services from the help desk. Support was limited to questions regarding the use and operation of the program. No self-management coaching was provided by any program resources."

5-xi) Report any prompts/reminders used

"Intervention group participants received messages weekly via e-mail reminding them to log in to MyHealth Online. Participants who fell below the desired threshold of participation (set at one log in per week) received a message tailored to this condition, encouraging them to increase their participation and to contact the help desk if they required assistance to use the application. All control group participants received a message midway through the study reminding them to review the health education material and to contact the CHS help desk with any questions."

"Each week of the study, intervention participants received an email alerting them to the availability of the next in a progressive series of health education sessions specific to their chronic condition."

5-xii) Describe any co-interventions (incl. training/support)

"All participants in the study had access to CHS' web site help desk for guidance on accessing and using the applications and to resolve technical problems. The intervention group participants were registered as end-users of MyHealth Online and receive no special services from the help desk. Support was limited to questions regarding the use and operation of the program. No self-management coaching was provided by any program resources."

6a-i) Online questionnaires: describe if they were validated for online use and apply CHERRIES items to describe how the questionnaires were designed/deployed

"Winsteps [52] was used to calculate the Rasch person reliability and infit statistics for assessing the PAM-13's reliability and validity. "

6a-ii) Describe whether and how "use" (including intensity of use/dosage) was defined/measured/monitored

"Research suggests that frequency of use of a web-based intervention – expressed as the number of user logins to the computer program – is associated with change in health outcomes the intervention is designed to influence [53, 65]. Adapting a method for analyzing frequency of use in a previous study of web-based interventions [53], the third hypothesis was tested by calculating the median value of cumulative logins during the study and using the result to divide intervention group participants into two subgroups."

6a-iii) Describe whether, how, and when qualitative feedback from participants was obtained

The significance of usability or user expectations to this study...is unknown, as measuring user satisfaction was not a study objective."

7a-i) Describe whether and how expected attrition was taken into account when calculating the sample size

Using a significance level of .05, a power of .8 and moderate effect size, the calculated target sample was 64 participants in each group at the start of the study before accounting for anticipated attrition. Attrition rates of 12% to 25% reported for RCTs involving health information technology were considered when establishing a planned 20% attrition factor for this study. Therefore, to increase the likelihood of the 128 sample size being maintained at the end of the study, each group contained at least 80 participants before commencement of the study.

7b) CONSORT

No interim analysis or stopping guidelines were part of this study.

8a) CONSORT

"Participants were randomly assigned to the intervention or control groups from pairs created by matching on adherence scores. A matching process was employed because it helps to mitigate differences between the groups at baseline and strengthens the study's statistical power [46]. The adherence scores were calculated based on participants' responses to four items in the enrollment questionnaire related to a person's adherence to self-management behavior. The two participants with the lowest adherence scores comprised the first pair and those with the highest scores the last. Starting at an arbitrary point in the stack of pairs, one member of the pair was assigned to the intervention and the other to the control."

8b) CONSORT

Refer to response in 8a) above.

9) CONSORT

Refer to response in 8a) above.

10) CONSORT

Participants enrolled online. The principal researcher executed the random allocation sequence and assigned participants to the intervention.

11a-i) Specify who was blinded, and who wasn't

"Participants were not informed of their intervention or control status."

Researchers were not blinded.

Support desk was not informed end-users were participating in a study.

11a-ii) Discuss e.g., whether participants knew which intervention was the "intervention of interest" and which one was the "comparator"

"Participants were not informed of their intervention or control status."

11b) CONSORT

Not relevant to this study.

12a) CONSORT

"...all statistical analysis was performed using PASW statistics release 18 programs [51]....Testing for differences between the two groups was conducted using the chi-square test of independence or Fisher's exact test for categorical variables and the t-test for independent groups for pre-test patient activation scores."

"For testing the primary hypothesis, the difference between groups in mean patient activation scores was evaluated by applying analysis of covariance (ANCOVA) using the mean patient activation score at pre-test as the covariate to reduce error variance. To test the second hypothesis, change in patient activation scores between participants in the intervention group starting at different stages of patient activation was analyzed using a two-step method. First, intervention participations were divided into three groups based on their stage of patient activation at the beginning of the study. These groups were treated as independent groups and one-way ANOVA applied to test for significant between groups. One-way ANOVA is appropriate for comparing means between three groups, but does not reveal the specific group differences underlying a significance difference [69]. To determine the specific groups (i.e., baseline stage) demonstrating significant change, a post hoc test, using Tukey's honestly significant difference method (HSD) was conducted."

"The independent samples t-test – appropriate for use in experimental studies to detect significant differences in mean scores between two groups [69] – was applied to test for differences in patient activation change scores between the high and low frequency groups. All inferential testing was two-tailed and conducted at the .05 level of significance."

12a-i) Imputation techniques to deal with attrition / missing values

"Characteristics of participants withdrawing or dropping out were analyzed to determine if any attrition-related factors needed to be controlled in the analysis."

"Testing for differences between the two groups was conducted using the chi-square test of independence or Fisher's exact test for categorical variables and the t-test for independent groups for pre-test patient activation scores."

Dropouts between groups were tested for differences; average pretest score of outcome measure of dropouts was compared to participants who completed the study. Dropouts and withdrawals were not included in the results of the study because they did not complete the posttest.

12b) CONSORT

Please refer to 12a)

RESULTS

13a) CONSORT

A participant flow diagram adhering to CONSORT guidelines appears in the manuscript (Figure 1).

13b) CONSORT

A participant flow diagram adhering to CONSORT guidelines, and including withdrawals and dropouts appears in the manuscript (Figure 1).

The control and intervention groups experienced attrition rates of 32% and 41% respectively by the end of the study (Figure 1). Characteristics of participants withdrawing or dropping out were analyzed to determine if any attrition-related factors needed to be controlled in the analysis.

"The six withdrawals and 24 dropouts may represent participants who experienced difficulties using the applications or had expectations for the web-based intervention that were not met. These are problems reported by users of web-based interventions in prior research [16]. The significance of usability or user expectations to this study's level of attrition is unknown, as measuring user satisfaction was not a study objective. "

13b-i) Attrition diagram

A participant flow diagram adhering to CONSORT guidelines, and including withdrawals and dropouts appears in the manuscript (Figure 1).

14a) CONSORT

This was a 12-week study conducted during the first-half of 2010.

14a-i) Indicate if critical "secular events" fell into the study period

No "secular events" occurred during the study period.

14b) CONSORT

The trial ended at the planned 12-week completion with a posttest.

15) CONSORT

Please refer to Table 1 and Table 2 in the manuscript.

15-i) Report demographics associated with digital divide issues

"The sample at baseline consisted of predominately non-Hispanic White persons between 45 and 64 years with a college degree. Slightly more women than men comprised the sample."

16-i) Report multiple "denominators" and provide definitions

Sample sizes are reported at the beginning and end of study for each group.
Cumulative use during the study, measured by logins is described.

16-ii) Primary analysis should be intent-to-treat

"At baseline no significant differences were found between the groups on any background variables, and the groups were not statistically different at the end of the study. Based on participants completing the study, there was no significant difference between the groups' mean pre-test patient activation scores. Thus, the two groups at posttest were not significantly different when the trial started."

17a) CONSORT

"Controlling for the pretest patient activation scores, a significant difference in posttest patient activation scores was found between the two groups ($F1, 123 = 4.438$, $P = .037$, effect size $r = .196$)."

"The one-way ANOVA revealed the mean change in patient activation scores across the three groups was significantly different, $F(2,55) = 6.472$, $P = .003$, effect size $r = .436$. The HSD test showed a significantly lower mean change score in the stage four group compared to the combined stage one-two group ($P = .014$) and participants starting at stage three ($P = .011$). The difference in change scores between the stage one-two and stage three groups was nonsignificant ($P = .999$)."

17a-i) Presentation of process outcomes such as metrics of use and intensity of use

"Patients logging into the web-based intervention at least 12 times during the twelve-week study (i.e., the high group) experienced a significantly larger change in patient activation scores compared to participants in the low group $t(56) = -2.862$, $P = .006$, effect size $d = .764$ (Table 5)."

17b) CONSORT

Binary outcomes are not applicable to this study.

18) CONSORT

No other analysis was performed in this study.

18-i) Subgroup analysis of comparing only users

Participants at the end of the study were not statistically significantly different from the groups at the start of the study. For inferential analysis of the results from an experiment, this is the important assumption that must be tested.

19) CONSORT

No harms or unintended effects were reported in this study.

19-i) Include privacy breaches, technical problems

No privacy breaches or technical problems occurred in this study.

19-ii) Include qualitative feedback from participants or observations from staff/researchers

No qualitative research was conducted as part of this study.

DISCUSSION

20-i) Typical limitations in ehealth trials

"Three limitations should be considered when interpreting the results. First, results may not be applicable to other populations and settings. Compared to the U.S. population [4], the sample under-represented people belonging to minority groups. Furthermore, 95% of the study participants had attended college. This study follows a persistent pattern of research involving W-SMIs, which attract mostly college-educated, non-Hispanic White participants [17,24,37,53,65,66]. Thus, applicability of results from most research of web-based interventions is inherently limited to a population willing and able to access the Internet. Second, the scope of this research was limited to measuring change in patient activation when a web-based intervention was used. The relative importance of specific functions and participants' perceived value of the web-based intervention were not assessed. A third limitation is the possibility that participants were influenced by agents external to the study. Participants were encouraged to receive usual care; no restrictions were placed on use of other support resources."

21-i) Generalizability to other populations

Please refer to above response to 20).

21-ii) Discuss if there were elements in the RCT that would be different in a routine application setting

No elements would be different in a routine application setting.

22-i) Restate study questions and summarize the answers suggested by the data, starting with primary outcomes and process outcomes (use)

"Results from this controlled trial suggest that a web-based intervention designed for use by patients with a variety of chronic medical conditions may improve the level of patient activation, a measure of self-management capabilities. A small but significant difference in posttest patient activation scores was found between the control and intervention groups. The small effect size found between the groups' patient activation scores is consistent with other web-based interventions demonstrating a significant effect on health behavior outcomes [23]. Within the intervention group, participants starting this twelve-week study at the first three stages of patient activation demonstrated significant improvement in their patient activation scores compared to patients who started at the most advances stage (four) of activation. Last, results from this study show a strong relationship between frequency of use of the web-based intervention and change in patient activation scores."

22-ii) Highlight unanswered new questions, suggest future research

Research in the interaction of web-based interventions and patient activation is needed in three areas. First, further study is warranted to validate the results from this study with larger samples that are more representative of the U.S. chronic disease population....Second, although this study showed an association between use of a web-based intervention and improvement in patient activation, the causal structure of this relationship is not well understood. Additional research is recommended to understand the cause and effect relationship between baseline patient activation levels, frequency of use of a web-based intervention, and change in patient activation. If frequency of use is established as a moderating variable [63], then strategies directed at sustaining use of the web-based interventions at optimal frequency levels will be critical to broad adoption of this technology.
Third, in-depth evaluation of the specific functions of web-based interventions is needed to discern which features are perceived as most beneficial to patients."

Other information

23) CONSORT

The protocol for this study was approved by the Carolinas Healthcare System Research Review Committee. A trial registry number was not obtained.

24) CONSORT

The full protocol approved by the CHS Research Review Committee is available from the CHS RRC office.

25) CONSORT

No external funding supported this study.

X26-i) Comment on ethics committee approval

"This study's research protocol was approved by the CHS Research Review Committee. Institution Review Boards of Walden University and CHS also approved the study."

x26-ii) Outline informed consent procedures

The consent procedure was conducted entirely online. Participants were directed to a CHS website to review the study and complete the consent form.

X26-iii) Safety and security procedures

Data was secured in accordance with procedures required by the CHS and Walden University IRBs. The consent form provided participants with contact information for IRB staff.

X27-i) State the relation of the study team towards the system being evaluated

The researchers have no relationship with the companies who developed the intervention and control materials.
